# Interactive effects of two rodent species on the seed dispersal of Japanese walnut

**DOI:** 10.1038/s41598-023-44513-9

**Published:** 2023-10-23

**Authors:** Ryunosuke Okawa, Takashi Saitoh, Takashi Noda

**Affiliations:** 1 Graduate School of Environmental Science, Hokkaido University, N10 W5, Kita-ku, Sapporo, Hokkaido 060-0810 Japan; 2 Field Science Center, Hokkaido University, N11W10, Kita-ku, Sapporo, Hokkaido 060-0811 Japan

**Keywords:** Biodiversity, Community ecology, Forest ecology

## Abstract

The effects of seed dispersers on plant fitness (seed dispersal effectiveness, SDE) have been evaluated based on the number (quantity) and recruitment probability (quality) of dispersed seeds. Although seeds of most zoochorous species are dispersed by two or more animal species, which may interact with each other, SDE has often been studied assuming a one-plant and one-animal species system. We compared the SDE of Japanese walnut (*Juglans ailanthifolia*) between squirrel-only and squirrel-mouse sites in natural forests of Hokkaido, Japan, and found that the SDE from the red squirrel (*Sciurus vulgaris*), considered a primary seed disperser, was altered by an alternative seed disperser species, the Japanese wood mouse (*Apodemus speciosus*). Seed removal rates at the squirrel-mouse site were significantly higher than those at the squirrel-only site, and both dispersed seeds and seedlings were less aggregated, with a strongly repulsive relationship with adult conspecific trees at the squirrel-mouse site. Seedlings established themselves at a location with fewer medium-sized trees (< 10 cm DBH) at the squirrel-mouse site. These results suggest that the interactive effect of the rodent species affects the SDE of Japanese walnut.

## Introduction

Animals play an important role as seed dispersers in the regeneration of many plant species^1, 2^. Seed dispersers may increase plant fitness by conveying seeds from a mother tree and depositing them in microhabitats favourable for survival or growth^3, 4^. The seed dispersal effectiveness (SDE) is estimated as the product of the number of seeds dispersed (quantity) and the probability of recruitment of each dispersed seed (quality)^3, 4^. The microhabitat of dispersed seeds and its spatial pattern have been the focus of previous studies on the qualitative component of SDE^3–5^. This is because spatial aggregation of seeds (seedlings) leads to sibling competition and reduces the reproductive probability of each individual^6^. Moreover, seeds and seedlings that settle near a conspecific adult tree are likely to be attacked by distance- or density-responsive pests and pathogens^7, 8^.

The seeds of most zoochorous species are dispersed by more than one animal species^9, 10^. Typically, their SDE has been independently evaluated for individual plant species and seed disperser species^5, 11^. Even when focusing on two or more animal species, SDE has been investigated as an independent pairwise plant-animal system without considering the interaction between seed dispersers. SDE calculated for isolated species might be inaccurate because multiple disperser species may interact with each other to affect seed fate under natural conditions^12, 13^. For example, rodents affect the fate of seeds that are dispersed by dung beetles. Beetles disperse seeds in dung deposits after endozoochory in warm temperate and tropical regions^14^. Dung beetles bury dung with seeds under the ground to prevent competitors such as rodents and other beetles from using the seeds. Although many of these seeds can survive until germination, rodents often remove and consume them when dung is not buried deeply^14, 15^. However, few studies have evaluated SDE considering the effects of multiple species of seed dispersers (but see refs. ^16, 17^ for exceptions).

The Japanese walnut (*Juglans ailanthifolia*) produces a very hard seed shell. After walnut seeds drop to the ground, the red squirrel (*Sciurus vulgaris*) and the Japanese wood mouse (*Apodemus speciosus*) disperse walnut seeds and hoard them under the ground in Hokkaido, Japan^18–20^. Red squirrels store each seed separately in the litter layer on the ground (scatter hoarding)^18, 19^. Wood mice also carry out scatter hoarding in the litter layer and occasionally store two or more seeds in the same burrows (larder hoarding)^18^. Wood mice are known to find seeds buried by red squirrels and steal them when both species coexist^19, 21^. Therefore, the SDE of red squirrels on the Japanese walnut may vary depending on the presence of wood mice.

This study aimed to elucidate the effect of interactions between red squirrels and Japanese wood mice on the SDE of the Japanese walnut. The specific objectives were: (1) to determine how the different combinations of seed dispersers affect the quantitative component of SDE; (2) to elucidate how the qualitative component of SDE, including the spatial distribution pattern of dispersed seeds and current-year seedlings (the degree of spatial aggregation within the seedling cohorts and spatial relationship between them and conspecific adult trees), varies depending on the different combinations of dispersers; and (3) to show how microhabitat characteristics of walnut seedlings differ between habitats with different combinations of dispersers. We tracked the dispersal of magnet-attached walnut seeds to compare the spatial distribution pattern (i.e., the spatial aggregation of walnut seeds and the spatial relationship between walnut seeds and adult conspecific trees) and removal rates of walnut seeds between the habitats in which both red squirrels and wood mice were present (the squirrel-mouse site) and those where only squirrels were present (the squirrel-only site). We also observed the dispersal of walnut seeds in a habitat without dispersers. Furthermore, we investigated the spatial distribution pattern of current-year seedlings and compared the microhabitat characteristics of seedlings between the squirrel-mouse and squirrel-only sites. In this study, we were not able to assess SDE from the wood mouse alone at a mouse-only site because we could not exclude red squirrels in the wild.

## Results

### Magnet-attached seed tracking

**Figure 1:**
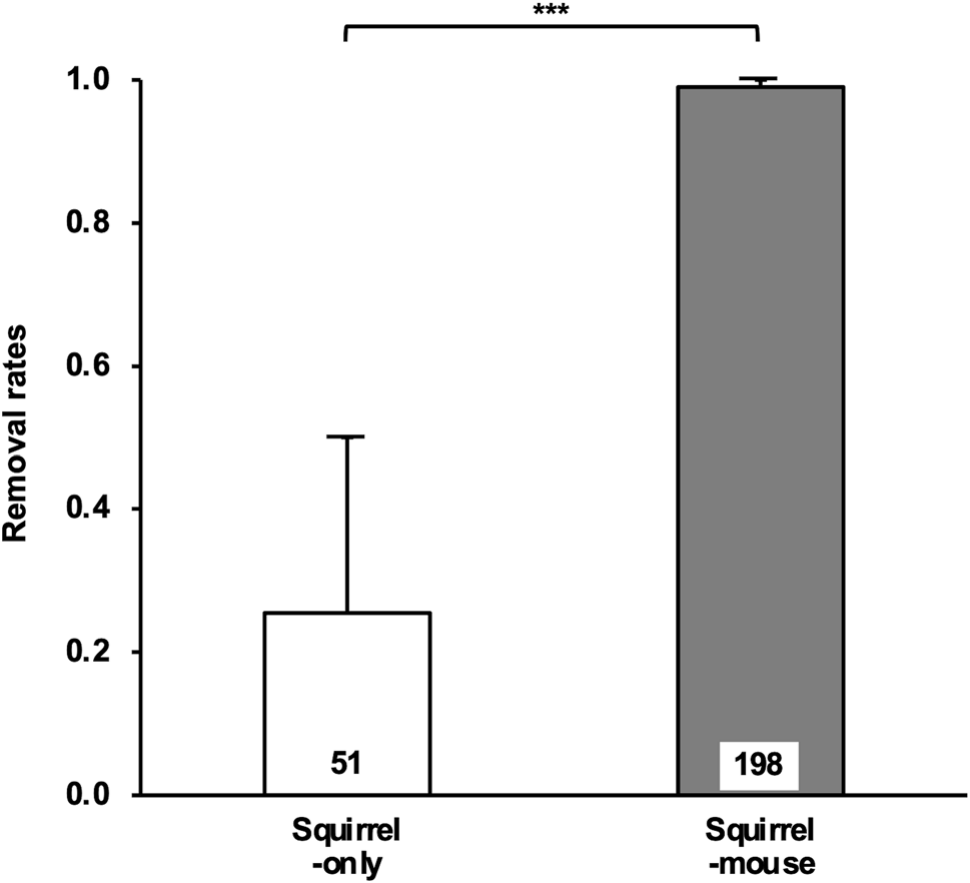
Removal rates of 200 magnet-attached walnut seeds placed at each squirrel-only and squirrel-mouse site. The figure shows the mean ± 1 SE. *** *p* < 0.001 indicates significant difference between two sites. The number of removed seeds is shown inside bars.

At the squirrel-mouse site, 198 (99%) out of 200 placed magnet-attached walnut seeds were relocated from the initial position. Seventeen (8.6%) of the relocated seeds were dispersed, three (1.5%) were eaten by mice, 59 (29.8%) were found with only the magnet intact (no walnut seeds), and 119 (60.1%) were not found. At the squirrel-only site, 51 (25.5%) out of 200 placed magnet-attached seeds were relocated from the initial position. Five (9.8%) of the relocated seeds were dispersed, 44 (86.3%) were found with only the magnet intact, and two (3.9%) were not found. The removal rates of magnet-attached seeds (the percentage of relocated seeds) were significantly higher at the squirrel-mouse site than at the squirrel-only site (chi-square test: *p* < 0.001; Fig. 1).

### Spatial pattern of dispersed seeds and current-year seedlings

**Figure 2:**
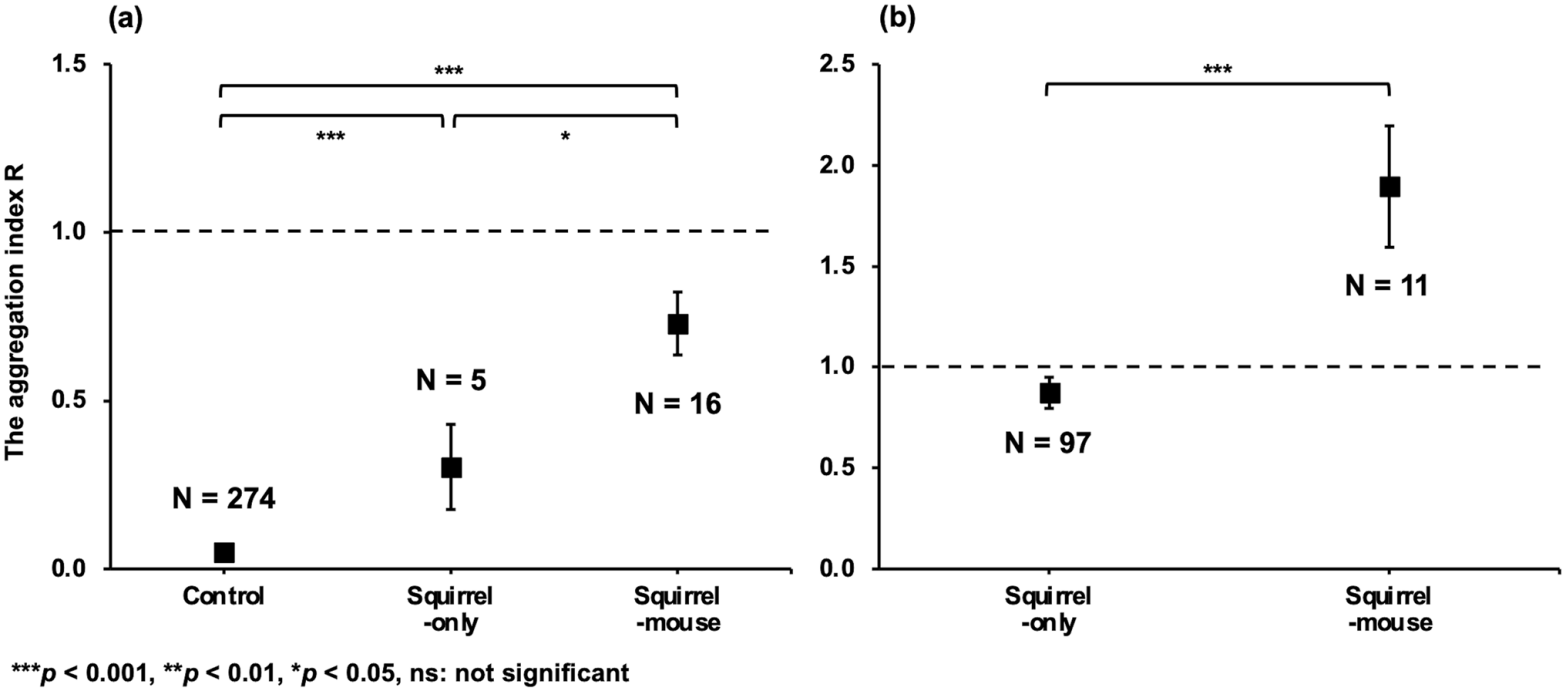
Result of Mann‒Whitney U test on the aggregation index R of dispersed magnet-attached seeds and gravity-dispersed seeds for the squirrel-mouse, squirrel-only and control sites (**a**) and current-year walnut seedlings for the squirrel-mouse and squirrel-only sites (**b**). The figure shows the mean ± 1 SE of the aggregation index R. R = 1 if the spatial pattern is random (dotted line), R < 1 when clumping occurs, and R > 1 indicates a uniform distribution pattern.

The spatial distributions of dispersed walnut seeds were significantly different among the sites with different combinations of dispersers. The aggregation index R was the largest at the squirrel-mouse site (i.e., the most uniform spatial distribution), followed by those at the squirrel-only site (Mann‒Whitney U test: *p* =0.023) and control site (i.e., site with no seed disperser; *p* < 0.001) (Fig. 2).

In total, 108 current-year walnut seedlings were found from the squirrel-mouse and squirrel-only sites. Spatial distributions of current-year seedlings were also significantly different between the squirrel-mouse and squirrel-only sites. The aggregation index R was larger at the squirrel-mouse site than at the squirrel-only site (Mann‒Whitney U test: *p* < 0.001; Fig. 2).

**Figure 3:**
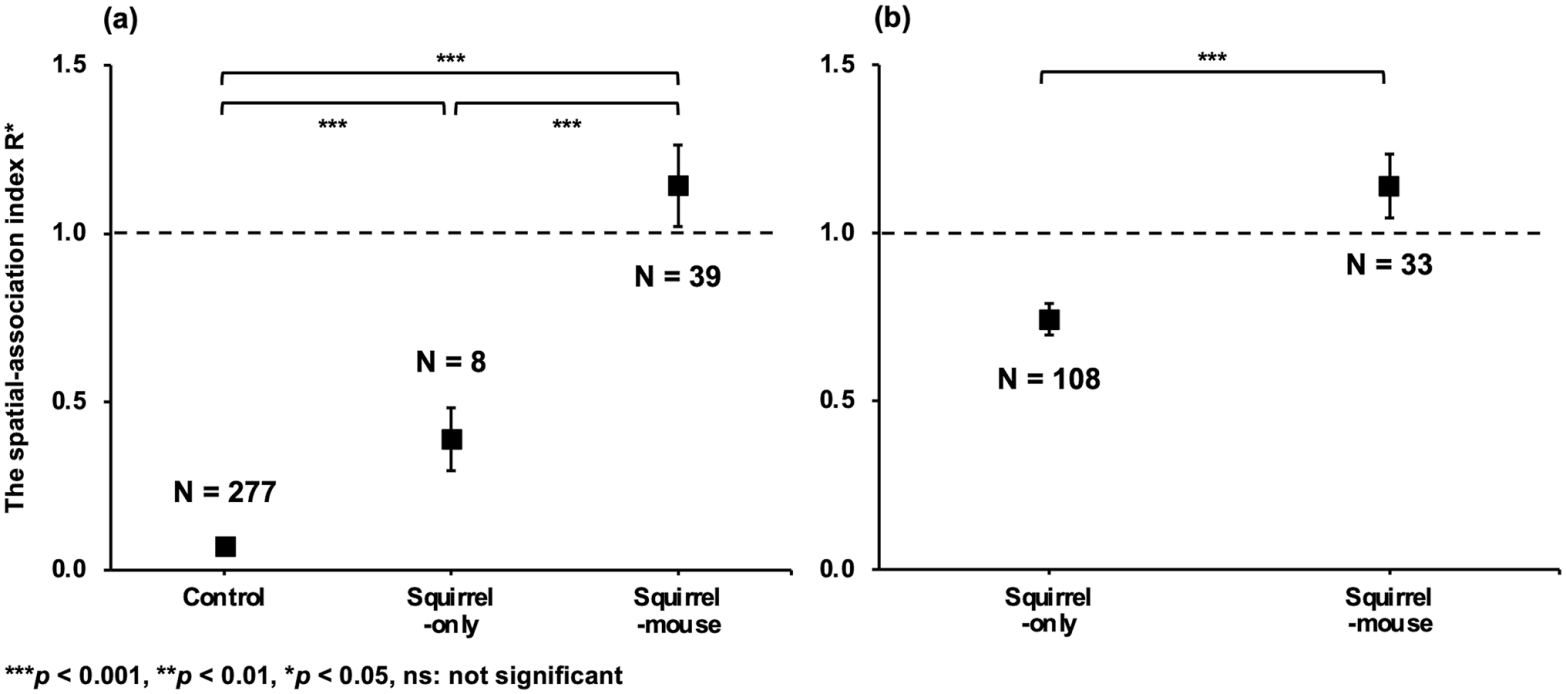
Result of the Mann‒Whitney U test on the spatial-association index R* between the adult walnut trees and dispersed magnet-attached seeds for the squirrel-mouse, squirrel-only and control sites (**a**) and current-year seedlings for the squirrel-mouse and squirrel-only sites (**b**). The figure shows the mean ± 1 SE of the spatial-association index R*. R* = 1 if the two distributions are independent (dotted line), R < 1 when there is an attractive relationship, and R > 1 when there is a repulsive relationship.

Spatial relationships between the adult walnut trees and dispersed seeds were significantly different among the sites with the different combinations of dispersers. The spatial-association index R* was largest at the squirrel-mouse site (i.e., the most repulsive relationship between seedlings and adult trees), followed by those at the squirrel-only (Mann‒Whitney U test: *p* < 0.001) and control sites (*p* < 0.001) (Fig. 3).

Spatial relationships between the adult walnut trees and the current-year seedlings were also significantly different between the squirrel-mouse and squirrel-only sites. The spatial-association index R* was larger at the squirrel-mouse site than at the squirrel-only site (Mann‒Whitney U test: *p* < 0.001; Fig. 3). Similar trends to these results were observed for both Ripley’s L(r) function and the cross-type Ripley’s L(r) function depending on distance (see Supplementary Results 1).

### Microhabitat of current-year seedlings

**Figure 4:**
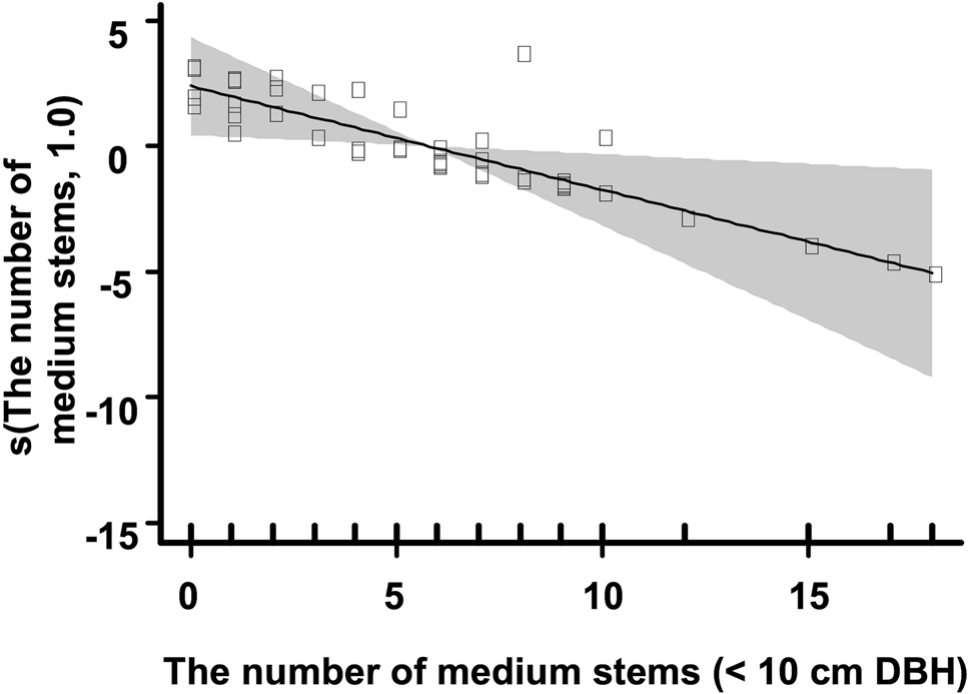
Output from the GAM illustrating the relationship (continuous line) between the occurrence of current-year walnut seedlings and the number of medium-sized trees (< 10 cm DBH) at the squirrel-mouse site. Standard errors (greyscale) are also shown. The *y* axis is a relative scale, with a positive *y* value on the plot indicating a positive effect of the number of medium-sized trees on the occurrence of the seedlings and a negative *y* value indicating a negative effect. The vertical lines in the plot indicate the number of medium-sized trees within 2 m from the location of seedlings and the centre of each grid inside the study site.

The results of the GAM revealed that the occurrence of walnut seedlings in the current year decreased with the increase in the number of medium-sized trees (< 10 cm DBH) at the squirrel-mouse site, although it was not dependent on any variable for microhabitats at the squirrel-only site (Table 1, Fig. 4).

**Table 1:** Summary of the results of generalized additive models for the occurrence of current-year walnut seedlings for each squirrel-only and squirrel-mouse site.

Squirrel-only site				Squirrel-mouse site			
Variables	Parametric coefficients			Variables	Parametric coefficients		
	Estimate	SE	*p* value		Estimate	SE	*p* value
(Intercept)	3.142	0.757	3.340e^−5^	(Intercept)	−1.625	0.583	0.005
Number of medium stems (< 10 cm DBH)	16.266	6.711 e^7^	1.000				

	Approximate significance of smooth terms				Approximate significance of smooth terms		
	edf	Ref. df	*p* value		edf	Ref. df	*p* value
Number of large stems (> 10 cm DBH)	1.000	1.000	0.364	Number of large stems (> 10 cm DBH)	1.000	1.000	0.312
Slope angle	1.000	1.000	0.271	Slope angle	1.000	1.000	0.343
Understorey coverage	1.001	1.001	0.762	Understorey coverage	1.000	1.000	0.846
Light availability	1.000	1.000	0.289	Light availability	2.674	3.359	0.230
				Number of medium stems (< 10 cm DBH)	1.000	1.000	0.014*

The number of medium-sized trees at the squirrel-only site was used as a linear function instead of a smooth function in the model because it had fewer unique covariate combinations than the specified maximum degrees of freedom. *p < 0.05

## Discussion

The removal rates of walnut seeds were significantly higher at the squirrel-mouse site than at the squirrel-only site (Fig. 1). Furthermore, the density of red squirrels (1.52–1.53 individuals/ha estimated from hit rates^22^) at the squirrel-mouse site was much lower than that of wood mice (27–32 individuals/ha; see Supplementary Methods 2 and Supplementary Results 2). These results suggest that wood mice largely contribute to the removal of walnut seeds at the squirrel-mouse site by removing many walnut seeds. However, there are two observations that support the idea that red squirrels might also have contributed to the dispersal of walnut seeds at the squirrel-mouse site. First, the mean dispersal distance of removed walnut seeds at the squirrel-mouse site (14.0 m; see Supplementary Results 3) was much longer than that reported for wood mice (6.2 m)^20^. Second, red squirrels may rely on walnuts at the squirrel-mouse site because the density of red squirrels at the squirrel-mouse site was not low compared with the forest in a similar climate region, even though there were no *Pinus koraiensis* trees, the main alternative diet of red squirrels^18, 23^ at the study sites. In addition, the density of red squirrels on the squirrel-mouse site (1.52–1.53 individuals/ha estimated from the hit rates) was unlikely to be below the density of the arboreal squirrels in the forest of Sila Grande Mountain (Italy^24^) and Hachioji City (Japan^25^), where these squirrels contributed to the seed dispersal of walnut species; Italian squirrels: 0.44 to 0.61 individuals/ha, Japanese squirrels: 0.06 to 0.13 individuals/ha.

The mean dispersal distance of walnut seeds by wood mice (6.2 m)^20^ is much lower than that of red squirrels (17.4 m)^19^. Therefore, if there was no interaction between wood mice and red squirrels, then dispersed walnut seeds would aggregate more clearly at the squirrel-mouse site than at the squirrel-only site. However, dispersed walnut seeds were less aggregated at the squirrel-mouse site (Fig. 2). These results suggest that the significant interaction between red squirrels and wood mice affects the spatial distribution pattern of dispersed walnut seeds at the squirrel-mouse site. Retrievals of walnut seeds by wood mice may explain this spatial pattern; wood mice may have retrieved walnut seeds that were dispersed by red squirrels and recached them^19, 21^. However, this hypothesis was not supported by the results of this study. The time interval from placing magnet-attached seeds to tracking them (approximately 3 weeks) was likely shorter than that of retrievals by mice (often occurring more than 3 weeks after caching^20^). Furthermore, the current-year walnut seedlings also exhibited less of a repulsive relationship with adult conspecific trees at the squirrel-mouse site than at the squirrel-only site (Figs. 2, 3), similar to the spatial pattern of dispersed seeds. This result suggests that the spatial pattern of dispersed seeds will not change significantly after initial dispersal. Although the spatial pattern of dispersed seeds can be modified by retrieval by wood mice or others until seedling germination, such effect was insignificant in this study. Alternatively, red squirrels may alter their hoarding behaviour to avoid the retrieval of buried seeds by wood mice. Rodents tend to disperse seeds in a location with a low seed density away from adult conspecific trees because places near conspecific trees have a high risk of the retrieval of cached seeds by competitors^26, 27^.

The sample sizes of the aggregation index R and the spatial-association index R* greatly varied across study sites (Figs. 2, 3). Although the statistical power of the test decreases as the inequality of sample sizes increases^28, 29^, significant differences were detected in our results (Figs. 2, 3), indicating the robustness of the differences. We assumed that the magnets that fell off during walnut seed tracking did not introduce bias in the estimate of the spatial pattern of the dispersed seeds. This is because the magnets likely fell off before dispersal because the distance between the locations where magnets without seeds were found and the initial position was significantly shorter than that of the dispersed magnet-attached seeds (Mann‒Whitney U test: *p* = 0.035 at the squirrel-only site, *p* = 0.003 at the squirrel-mouse site; see Supplementary Results 3).

In general, arboreal squirrels prefer to hoard seeds in open habitats with low tree canopy coverage^30, 31^, while wood mice prefer a closed habitat in which the light availability is low and the coverage of the understorey and tree density are high^32, 33^. Therefore, if there was no interaction between wood mice and red squirrels, then walnut seedlings in the current year would tend to be established in a closed habitat characterized by low light availability, a high density of trees, and high understorey coverage at the squirrel-mouse site compared to the squirrel-only site. However, seedlings tended to be established in a more open habitat at the squirrel-mouse site than at the squirrel-only site. This is because many seedlings were established at the microhabitat with fewer medium-sized walnut trees (< 10 cm DBH) at the squirrel-mouse site, whereas they did not exhibit such a pattern at the squirrel-only site (Table 1; Fig. 4). This result indicates that the interaction between red squirrels and wood mice affected the microhabitat use of the current-year walnut seedlings at the squirrel-mouse site. Walnut seeds dispersed by red squirrels to the microhabitat with many medium-sized trees may have been retrieved by wood mice. Wood mice favour a closed habitat and often retrieve and consume seeds cached by squirrels^19, 21^. Another possible mechanism is that red squirrels cache seeds selectively in microhabitats with fewer medium-sized trees to avoid the retrieval of buried seeds by wood mice. Arboreal squirrels tend to hoard seeds in an open habitat where the pilferage rates of hoarded seeds are low^30, 31^.

The results of this study suggest that the interaction between red squirrels and wood mice, in which mice may retrieve seeds from the caches of squirrels and consume them^19, 21^, improves the fitness of Japanese walnut. First, the risk of sibling competition and distance- or density-responsive pests and pathogens, major factors contributing to seedling failure^6–8^, decreases. This is because the dispersed walnut seeds and the current-year seedlings had a uniform distribution between the cohorts and had a repulsive relationship between the cohorts and adult conspecific trees at the squirrel-mouse site compared to the squirrel-only site. In addition, interspecific competition, considered a major cause of seedling failure for nut-producing plants^34^, decreases. This is because seedlings were established at the site where the number of medium-sized trees (< 10 cm DBH) was few at the squirrel-mouse site.

Our results provide important insights that SDE may not be properly evaluated unless considering the interactive effects of disperser species. Nonetheless, our study has two significant limitations. First, the adopted SDE framework was incomplete because we were not able to consider the SDE from the wood mouse alone, i.e., the mouse-only site. The SDE of the Japanese walnut can be predicted to be lower in the mouse-only site than in other sites because wood mice are known to have a lower dispersal ability of Japanese walnut seeds than red squirrels^19, 20^. However, both quantitative and qualitative components of SDE are context-dependent and vary with various conditions^34, 35^. Therefore, it cannot be logically ruled out that the results observed at the squirrel-mouse site were simply due to seed dispersal by wood mice rather than the interactive effect with red squirrels. The second limitation is that factors other than the combinations of seed dispersers may have influenced the results. For example, it is known that seed disperser density affects the fate of seeds^36–38^. However, there was no significant difference in the hit rates of camera traps for red squirrels between the squirrel-mouse and squirrel-only sites (t test: *p* = 0.317; see Supplementary Methods 2 and Supplementary Results 2). In addition, the density of adult walnut trees at the squirrel-mouse site was significantly higher than that at the squirrel-only site (*t* test: *p* = 0.025; Table 2). However, the difference in the density of the adult walnut trees should not have affected the current removal rates of walnut seeds. In general, the removal rates of seeds increase with an increasing ratio of seed disperser density to conspecific adult tree density^38, 39^. Nevertheless, this tendency is not in agreement with the expectation based on the general pattern. Although considerable differences in other conditions between the sites (e.g., numbers of large- and medium-sized trees; Table 2) may have affected the seed dispersal behaviour of dispersers, attention was not paid to those effects.

**Table 2:** Summary of five variables from data collected at the centre of each grid as representative characteristics of the entire study area, and the densities of walnut trees and dispersers at the squirrel-mouse and squirrel-only site, with values presented as the mean ± 1 SE.

Study site characteristics	Squirrel-mouse	Squirrel-only
Number of large stems (> 10 cm DBH) (individuals/m^2^)	0.041 ± 0.029	0.018 ± 0.021
Number of medium stems (< 10 cm DBH) (individuals/m^2^)	0.57 ± 0.39	0
Slope angle (°)	14.23 ± 0.82	23.0 ± 8.30
Understorey coverage (%)	30.19 ± 18.63	29.81 ± 26.43
Light availability (%)	5.96 ± 0.94	6.8 ± 2.04
Density of walnut trees (individuals/ha)	11.50 ± 6.36	2.67 ± 1.53
Hit rates of red squirrels (videos/camera-day)	0.031 ± 0.0087	0.046 ± 0.013
Density of wood mice (individuals/ha)	29.50 ± 3.54	–

## Conclusions

The SDE of Japanese walnut varied among the study sites with the different combinations of seed dispersers. The removal rates of seeds at the squirrel-mouse site were significantly higher than those at the squirrel-only site, and both dispersed seeds and seedlings were less aggregated with the strongly repulsive relationship with adult conspecific trees at the squirrel-mouse site. Seedlings established themselves at locations with fewer medium-sized trees (< 10 cm DBH) at the squirrel-mouse site, whereas they did not show any preference for microhabitat characteristics at the squirrel-only site. The results of this study suggest that the interactive effect of seed dispersers may affect plant fitness through the qualitative component of SDE and indicate that SDE, which has been evaluated in one plant species and one seed disperser species system^5, 11^, should be revised by considering knowledge from multiple species systems for seed dispersers. In an interactive system, a species that has been considered an inferior disperser may play an important role in improving plant fitness through interaction with other disperser species. Although wood mice have been considered a minor seed disperser for Japanese walnut^19, 21^, the results of this study suggest that they may alter the fitness of Japanese walnut through interaction with red squirrels.

## Materials & methods

### Study species

The Japanese walnut (*Juglans ailanthifolia*) is a deciduous tree species (height: up to approx. 20 m) and common in Hokkaido, Japan. It often occurs in early successional habitats, especially in flat or mild slope areas of riparian forests^40^. Walnut seeds have extremely hard shells protecting embryos measuring 1.5 to 4.3 cm in shell length and 4.3 to 14.8 g in fresh weight^41^. They drop to the ground in autumn, October to early November, as the primary means of dispersal and then are conveyed by the limited species of vertebrates from their natal site as the secondary means of dispersal^25, 42^. Japanese walnut seeds were identified using a field guide because they have highly characteristic morphology and are straightforward to identify.

In Hokkaido, walnut seeds are consumed primarily by two species of rodents: the red squirrel (*Sciurus vulgaris*) and the Japanese wood mouse (*Apodemus speciosus*)^18, 19^. The body weight of red squirrels is 350 to 456 g, while that of wood mice is 20 to 60 g^43^. Both species contribute to walnut seed dispersal, but each has a different hoarding behaviour that may affect walnut fitness. Although both red squirrels and wood mice carry out scatter hoarding, wood mice occasionally also carry out larder hoarding^18, 19^. Some scatter-hoarded seeds are not retrieved and can germinate. The germination rate of larder-hoarded seeds decreases with increasing hoarding depth (80% at 50 mm to 13% at 300 mm in depth^44^). Squirrels can hoard walnut seeds relatively far from the source tree than wood mice (red squirrels: mean 17.4 m; wood mice: mean 6.2 m^18, 19^). The survival rates of dispersed walnut seeds and seedlings are generally lower near the source tree and increase rapidly with increasing distance from the source tree^45^. Therefore, the natural dispersal of Japanese walnuts is thought to be primarily performed by red squirrels.

### Study sites

**Figure 5:**
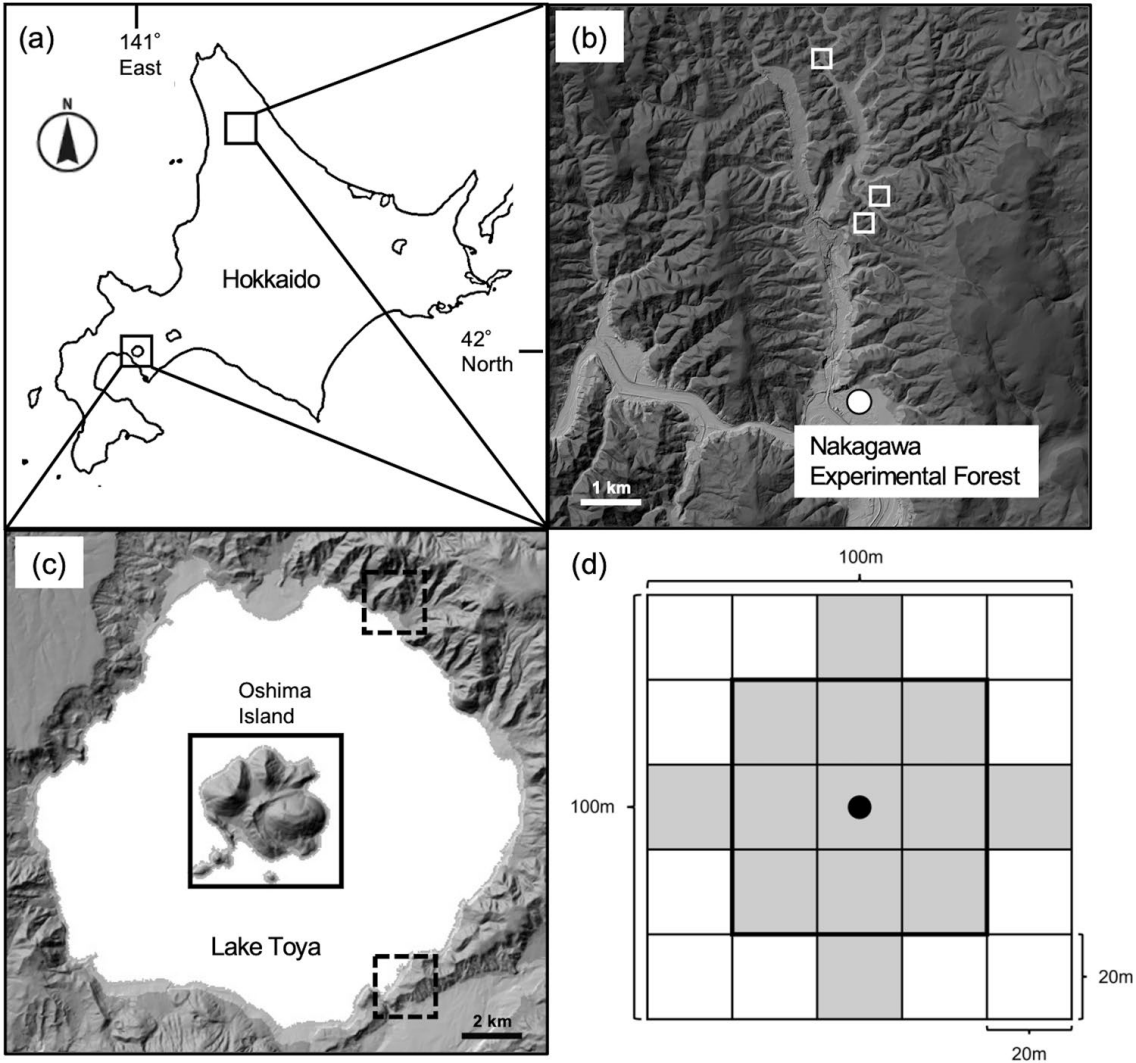
(**a**) Map of Hokkaido showing the position of Lake Toya. (**b**) Map of Nakagawa Experimental Forest showing three study sites. (**c**) Map of Lake Toya showing three study sites: two squirrel-mouse sites (dashed square) and one squirrel-only site (solid square). (**d**) Plot design established at each study site near Lake Toya. The black point in the panel represents the walnut tree growing at the centre of the plot. The grey squares indicate the range searched for current-year walnut seedlings. The area inside the dark line shows the range searched for magnet-attached walnut seeds. The satellite map is modified from the Digital Japan Portal Web Site, Geospatial Information Authority of Japan (http://maps.gsi.go.jp/).

We investigated the dispersal of walnut seeds by gravity in the Nakagawa Experimental Forest (142°15′ E, 44°20′ N), northern Hokkaido, Japan (Fig. 5a, b). Three study sites (hereafter the control sites) were selected where adult walnut trees (> 10 cm DBH, range 20.7–35.67 cm) grew separately; there were no other walnut trees within a radius of 15 m from them. The intervals between neighbouring sites ranged from 35 to 2600 m. The tree height and the mean canopy diameter of the observed walnut trees at each site varied from 8.34 to 14.88 m and 9.48 to 12.03 m, respectively.

Other investigations were conducted at three sites in mixed forests near Lake Toya (140°51’ E, 42°36’ N), southwestern Hokkaido, Japan, with the intervals between neighbouring sites ranging from 5.1 to 8.8 km (Fig. 5a, c). One study site (hereafter the squirrel-only site) had only red squirrels and was in Oshima Islet (497.8 ha), which is an islet in Lake Toya. At this site, the dominant canopy trees were deciduous broad-leaved trees, mainly *Acer mono*, *Tilia japonica*, and *Cercidiphyllum japonicum*, and the understorey was dominated by *Senecio cannabifolius* and *Pachysandra terminalis*. The other two study sites (referred to as the squirrel-mouse sites) had both red squirrels and wood mice and consisted of mixed deciduous forests (8.6 ha and 41.2 ha) on the shore of the lake. At both squirrel-mouse sites, the dominant canopy trees were *Acer mono*, *Quercus crispula*, and *Kalopanax septemlobus*, and the understorey was dominated by *Senecio cannabifolius* and *Sasa veitchii*.

Within each site, we established 2 (for the squirrel-mouse site) to 3 (for the squirrel-only site) 1.0-ha square plots whose centre was located on an adult walnut tree under which walnut seeds with bite marks showing vertebrate eating were observed (Fig. 5d). The intervals between neighbouring plots ranged from 200 to 1200 m. The angles of the slopes in the plots varied between 1.0° and 40.0° (mean ± SD: 20.0 ± 9.9°) from horizontal (0°).

### Gravity dispersal of walnut seeds

To investigate walnut seed dispersal by gravity, we measured the location of seeds dropping from the source tree using a 400-m^2^ sheet-shaped seed trap at each control site from the end of September to October 2018. At each site, the understorey within a 15 m radius from the walnut tree was removed with a grass cutter, and a seed trap was placed on the ground whose centre was located on the walnut tree. The seed trap was divided into 100 grids (2 × 2 m = 4 m^2^) to facilitate measuring the location of walnut seeds. We surrounded the periphery of each seed trap with a 1.0 m high net to prevent any terrestrial vertebrates from intruding into the seed trap. The angles of the slopes in the traps vary between 0.98° and 7.22° (mean ± SD: 3.39 ± 2.05°).

After placing seed traps, we searched for walnut seeds dropped from the central walnut tree inside the seed trap every five days until we could not find any new seeds and measured their *x* and *y* coordinates in each grid with a measuring tape.

### Seed tagging & tracking

We investigated walnut seed dispersal on squirrel-only and squirrel-mouse sites using magnet-attached walnut seeds. Walnut seeds were collected near each study site in May 2019 and soaked in water to discern sound seeds from empty ones. Sound walnut seeds varied in size (4.3–14.8 g in fresh weight^41^). We selected 400 average-sized seeds for experiments (${\overline{\text{X}}}$ ± SD = 5.92 ± 0.99 g, N = 1000) and attached a small ferrite magnet (3.5 mm in diameter × 3.0 mm in length, 0.22 g, 4300 G) on the shell of each walnut seed using a waterproof adhesive.

One hundred magnet-attached seeds were placed on the ground in two plots of each of the squirrel-only and squirrel-mouse sites in October 2019; 25 of the 100 seeds were placed 5 m away from the central walnut tree (the black point in Fig. 5d) in each of four directions. Then, we divided each plot into 25 grids (400 m^2^) to facilitate measuring the location of dispersed seeds (Fig. 5d). Three weeks after placing magnet-attached walnut seeds, we tracked them using a magnetic locator (GA-1, Fuji Tecom Inc., Tokyo, Japan) inside the nine grids (a 0.36-ha square area surrounded by the thick bold line in Fig. 5d) of each plot. We identified their locations, measured the distance between the position of recovered magnet-attached seeds and the initial position, and observed their states (dispersed, eaten, only magnet recovered, or not found).

### Seedling searching and microhabitat measurement

We located current-year walnut seedlings at the squirrel-mouse and squirrel-only sites from June to July 2019 to test the effects of seed dispersers on the spatial distribution patterns of seedlings. We searched for current-year seedlings by walking at ordinary speed (approx. 2-3 km h^−1^) inside the 13 grids (a 0.52-ha grey area in Fig. 5d) and for adult walnut trees (> 10 cm DBH) inside the whole plot. Then, we determined their locations in each grid based on the distance and direction from the upper left corner of the grid measured with a laser rangefinder (Tru-Pulse 200, Laser Technology Inc., Centennial, Colorado, USA).

We quantitively evaluated the microhabitat characteristics of current-year walnut seedlings at the squirrel-mouse and squirrel-only sites. For every seedling, we measured a series of variables that were selected based on their potential importance for seed fates based on the results of previous studies^46, 47^: (1) the number of large-sized trees (> 10 cm DBH) within 5 m; (2) the number of medium-sized trees (< 10 cm DBH) within 2 m; (3) the slope angles; (4) the understorey coverage; and (5) the percentage of canopy cover (light availability). To test the selectivity of seedlings with respect to microhabitat characteristics, we also measured the above five variables at the centre of each grid as representative characteristics of the entire study area. The understorey coverage was estimated by the Braun-Blanquet scale based on the eight abundance classes: 0, r, +, 1, 2, 3, 4 and 5 corresponding to the absence of the understorey, negligible cover, less than 0.1% cover, between 0.1 and 5% cover, between 5 and 25% cover, between 25 and 50% cover, between 50 and 75% cover, and more than 75% cover, respectively^48^. To determine the percentage of canopy cover, we took hemispherical photographs and calculated canopy cover using the CanopOn 2 program (http://takenaka-akio.org/etc/canopon2/index.html).

### Statistical analysis

To test the difference in the removal rates of magnet-attached walnut seeds between the squirrel-mouse and squirrel-only sites, the Chi-square test of independence was applied.

We evaluated spatial aggregation within the cohorts (current-year walnut seedlings, dispersed magnet-attached seeds or gravity dispersed seeds) using the aggregation index R^49^ for each plot. The aggregation index R is based on the measurement of nearest neighbour distances for each individual and provides an indication of whether the observed distribution has a clumped or uniform distribution compared to the expected random pattern. To assess the spatial relationship between the adult walnut trees and the current-year seedlings, dispersed magnet-attached seeds or gravity dispersed seeds, we used the spatial-association index R*^50^. The spatial-association index R* is based on the measurement of the nearest neighbour distance from points of one type to the other type and provides an indication of whether two distributions have an attractive or repulsive spatial relationship compared to an independent spatial association. We also used Ripley’s L(r) function and the cross-type Ripley’s L(r) function as complementary tests for the aggregation index R and the spatial-association index R*, respectively (see Supplementary Methods 1).

We used spatstat, an R package for spatial point pattern analysis^51^, to calculate the aggregation index R and the spatial-association index R* based on the *x*- and *y*-coordinates of each adult walnut tree, current-year seedling, dispersed magnet-attached seed and gravity dispersed seed for all plots. Regarding the aggregation index R and the spatial-association index R*, the Mann‒Whitney U test with Holm correction was used to test for significant differences among the squirrel-mouse, squirrel-only and control sites (i.e., site without seed disperser).

To test the effects of microhabitat characteristics on the occurrence of current-year walnut seedlings, we fitted a generalized additive model (GAM)^52^ with binomial error structure and logit-link function for each of the squirrel-mouse and squirrel-only sites, in which the dependent variable was whether the point was the location of current-year seedlings or the centre of each grid inside the study site (the occurrence of the seedlings), and the independent variables were the number of large-sized trees (> 10 cm DBH), the number of medium-sized trees (< 10 cm DBH), angles of the slope, understorey coverage, and light availability. GAM was used, rather than a more conventional linear modelling approach, because the expected relationships are likely to be nonlinear. Some independent variables were significantly different between the squirrel-mouse and the squirrel-only sites, and therefore, we fitted the GAM to each site separately. Among the independent variables, the number of medium-sized trees at the squirrel-only site had fewer unique covariate combinations than the specified maximum degrees of freedom. Thus, we used a linear function for this variable instead of a smooth function in the model. The variance inflation factor (VIF) was assessed to determine the potential multicollinearity among the independent variables for each site^53^. The VIF among the independent variables ranged from 1.04 to 1.56, indicating negligible multicollinearity among the independent variables.

### Ethics statement

The field research in the Nakagawa Experimental Forest was permitted by the Nakagawa Experimental Forest of the Field Science Center for Northern Biosphere of Hokkaido University. Permission to perform the study, including permission to collect seeds and catch small mammals at Lake Toya, was issued by the Shiribeshi District Forest Office of the Forestry Agency and the Iburi Subprefecture of the Hokkaido Government (18-0114). All methods employed in this study were performed in accordance with the institutional, national and international guidelines and regulations.

### Supplementary Information

Supplementary Information.

## Data Availability

The datasets used and/or analysed during the current study are available from the corresponding author on reasonable request.
