# Supplementary material for: Interactive effects of two rodent species on the seed dispersal of Japanese walnut
Source: Sci Rep. 2023 Oct 23;13:18098. doi: 10.1038/s41598-023-44513-9 (PMC10593932; doi:10.1038/s41598-023-44513-9)
Supplement: Supplementary file 1 — Supplementary Information. [file 41598_2023_44513_MOESM1_ESM.pdf]

Supplementary information for "Interactive effects of two rodent species on the seed dispersal of Japanese walnut"

Ryunosuke Okawa[1], Takashi Saitoh[2], Takashi Noda[1]

[1] Graduate School of Environmental Science, Hokkaido University, N10 W5, Kita-ku, Sapporo, Hokkaido 060-0810, Japan.

[2] Field Science Center, Hokkaido University, N11W10, Kita-ku, Sapporo, Hokkaido 060-0811, Japan.

*corresponding author, contact: r.okawa44@gmail.com

Supplementary Methods 1

Ripley's L(r) function and the cross-type Ripley's L(r) function are methods used to complement the aggregation index R and the spatial-association index R*, respectively. Because these functions and indexes have different characteristics (Ripley's L(r) and cross-type Ripley's L(r) are cumulative, while the aggregation index R and spatial-association index R* are not), it is appropriate to use them together to derive the most information from the pattern[1,2]. Ripley's L(r) is a transformation of Ripley's K(r) to determine the expected number of neighbours in a circle of radius r centred on an arbitrary point in the point pattern[3,4]. This circle begins at a specified radius and is increased until it encompasses the entire study area. Ripley's K(r) is defined so that $\lambda K(r)$ equals the expected number of additional points of the spatial pattern resulting from a random point process, X, within a distance r of a point of X, where λ is the intensity (expected number of points per unit area), as in the equation:

K(r)=πr^2

To attain constant variance and easier interpretation of the results, Ripley's K(r) is often transformed into Ripley's L(r):

L(r)=√(K(r)⁄π)

The cross-type Ripley's L(r) is a generalization of Ripley's L(r) for a categorical marked point pattern and shows the expected standardized number of events of one type (e.g., adult trees) for the distance r from the events of the other type (e.g., current year seedlings, dispersed magnet-attached seeds or gravity dispersed seeds)[4,5].

We used spatstat, an R package for spatial point pattern analysis[4,] to calculate the Ripley's L(r) and the cross-type Ripley's L(r) based on the x, y coordinate of each adult walnut tree, current year seedling, dispersed magnet-attached seed and gravity dispersed seed for all plots. Regarding Ripley's K(r) and the cross-type Ripley's L(r), we estimated confidence intervals using the Monte Carlo method[6]. Nineteen heterogeneous Poisson patterns were simulated as the null model, and the confidence interval was defined as the 5% lowest and highest values of

Ripley's L(r) and the cross-type Ripley's L(r) at distance r[7,8]. The spatstat package was also employed for all calculations of Ripley's L(r) and the cross-type Ripley's L(r) with the adjustment of edge effects using the isotropic method[4].

Supplementary Methods 2

We conducted camera trapping using infrared cameras (Moultrie Digital Game Camera, model D-555i, M-1100i) to estimate the density of red squirrels at the squirrel-only and squirrel-mouse sites from September to November 2019. The cameras were set to record a 60-s video when triggered, and the delay option was set to the lowest possible setting for all cameras (videos could be recorded as little as 5 s apart). The motion sensitivity in all cameras was set to high. Camera trapping sessions were 3 days long and involved 18 cameras placed on each 1.0-ha plot where we conducted magnet-attached seed tracking. We secured each camera to a tree 1 to 3 m from the corner of the grid and approximately 10 to 30 cm above the ground such that the intervals between neighbouring cameras were 28 m. Then, we placed peanut butter and sunflower seeds in front of each camera on the ground to bait red squirrels at the beginning of the camera trapping session. The density of red squirrels was estimated for each plot using hit rates; the total number of videos of red squirrels obtained during the camera trapping session by 18 cameras divided by the total effort in camera days (the number of 24-h periods each camera was operational for during that filming session). If consecutive videos of individuals occurred within 5 min, we grouped them as a single hit to avoid recounting the same individual[9].

To estimate the density of wood mice, we conducted live trapping at the squirrel-mouse site from September to October 2019. We placed 61 live traps (The Sherman Traps, 23 × 9 × 7.5 cm) on the ground in each 1.0-ha plot where we conducted magnet-attached seed tracking such that the intervals between neighbouring traps were 14 m. Each live trapping session was 3 days long. At the beginning of the live trap session, all traps were baited with sunflower seeds and oats. Afterwards, we checked the live traps and rebaited them every morning. Captured wood mice were tagged

using permanent markers, and their mass and sex were recorded. Wood mouse densities were estimated using the number of individuals captured during the live trapping session (minimum number alive or MNA) divided by plot area.

Supplementary Results 1: Spatial aggregation

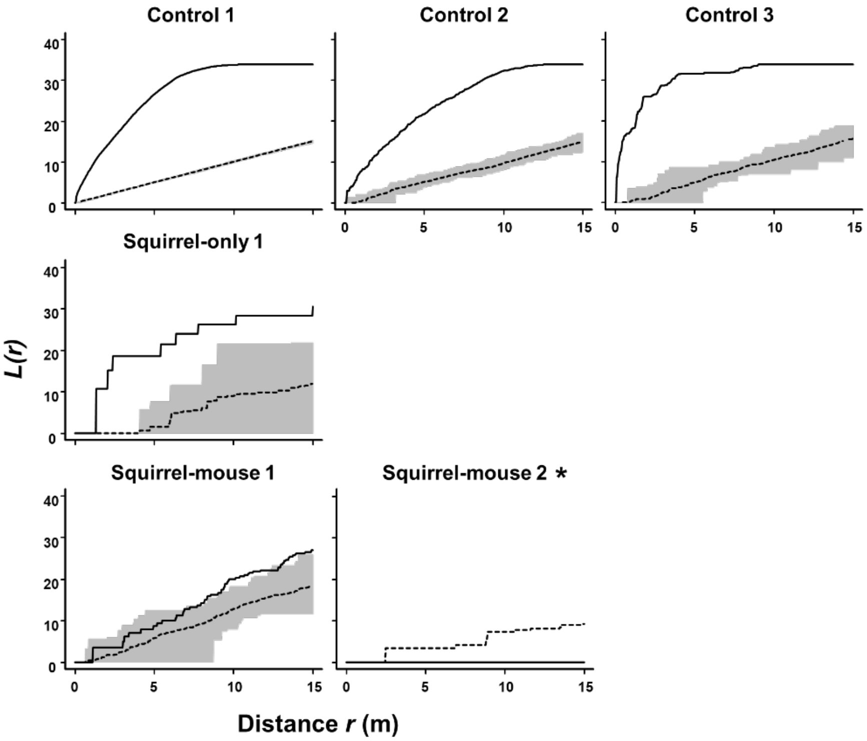

Supplementary Figure 1. The spatial aggregation statistic for dispersed magnet-attached walnut seeds and gravity-dispersed seeds, L(r), evaluated at a range of distances r for each plot of the squirrel-only, squirrel-mouse and control sites. When L(r) (continuous line) is outside the confidence interval (with a risk of α = 0.05; greyscale), it indicates that there is evidence against the null model (heterogeneous Poisson pattern; dotted line). Measured values below the confidence interval indicate a uniform pattern, and values above the interval indicate a clumped pattern.

* The confidence interval was not obtained because of the small sample size (N = 2)

Ripley's L(r) confirmed a clumped distribution in all plots excluding one plot of the squirrel-mouse site. Whereas the control and squirrel-only sites exhibited a clumped distribution up to different distances r, a significant departure from spatial randomness was found in the limited range (more than 10 m in distance r) in one plot of the

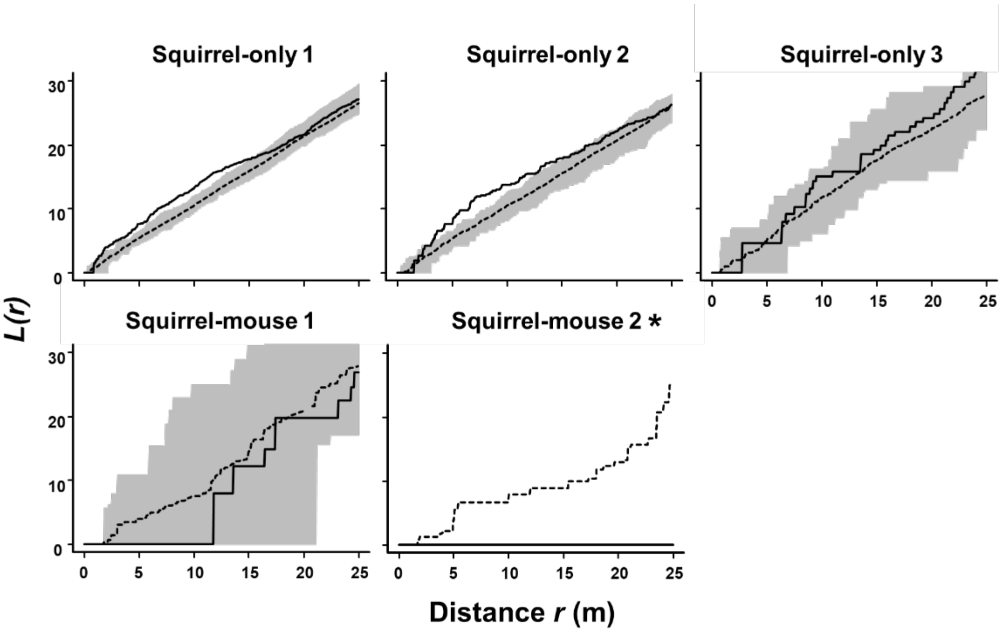

squirrel-mouse site.

Supplementary Figure 2. The spatial aggregation statistic for the current year walnut seedlings, L(r), evaluated at a range of distances r for each plot of the squirrel-mouse and squirrel-only site. When L(r) (continuous line) is outside the confidence interval (with a risk of α = 0.05; greyscale), it indicates there is evidence against the null model (heterogeneous Poisson pattern; dotted line). Measured values below the confidence interval indicate a uniform pattern, and values above the interval indicate a clumped pattern.

* The confidence interval was not obtained because of the small sample size (N = 3)

Although the departure from randomness was not statistically evident in one plot of the squirrel-only and squirrel-mouse sites, there was a tendency for the value of Ripley's L(r) for seedlings to fall above the mean value obtained from the null model in all plots of the squirrel-only site but fell below in all plots of the squirrel-mouse site.

Supplementary Results 1: Spatial association

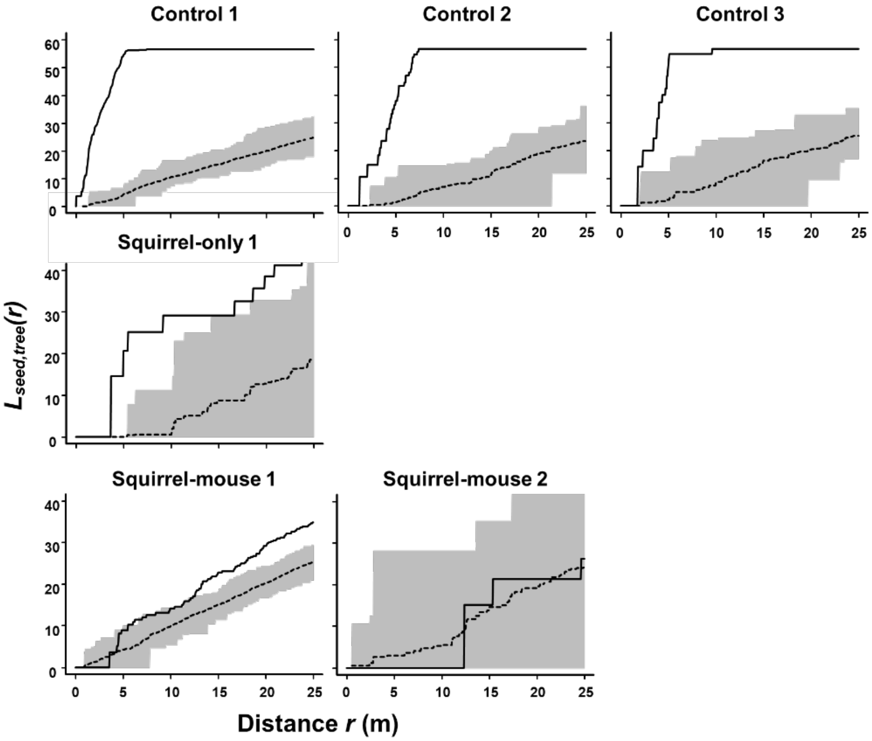

Supplementary Figure 3. The spatial association statistic between the adult walnut trees and dispersed magnet-attached seeds or gravity dispersed seeds, cross-type L(r), evaluated at a range of distances r for each plot of the squirrel-mouse, squirrel-only and control site. When cross-type L(r) (continuous line) is outside the confidence interval (with a risk of $\alpha = 0.05$; greyscale), it indicates there is evidence against the null model (heterogeneous Poisson pattern; dotted line). Measured values below the confidence interval indicate a repulsive relationship, and values above the interval indicate an attractive relationship.

The cross-type Ripley's L(r) for dispersed seeds confirmed an attractive spatial relationship with adult trees in all plots excluding one plot of the squirrel-mouse site. Although the control and squirrel-only sites showed an attractive spatial relationship between dispersed seeds and adult trees up to different distances r, the departure from independence was confirmed more than 15 m in distance r in one plot of the squirrel-mouse site.

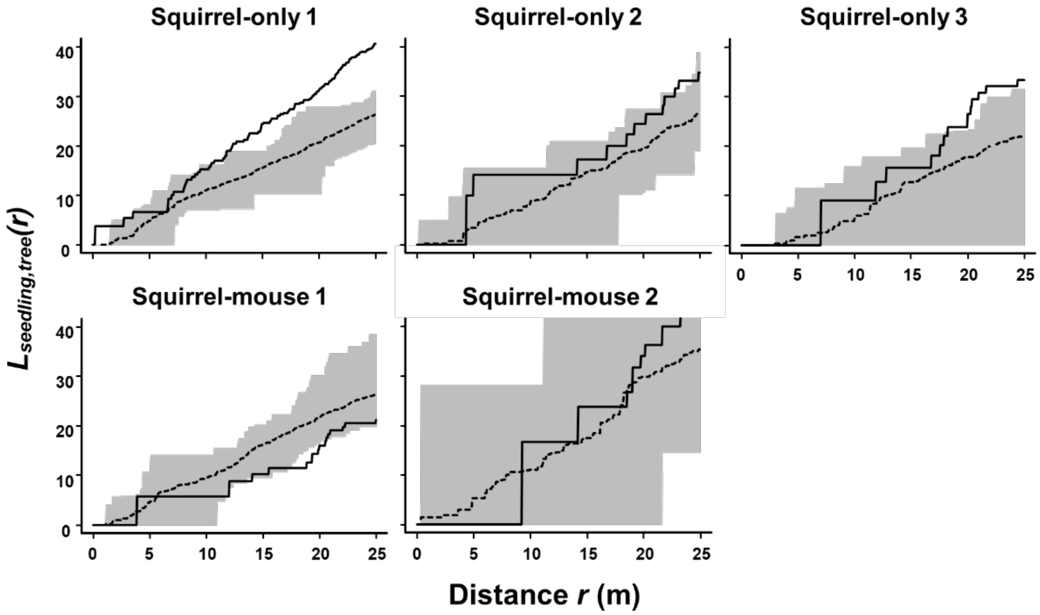

Supplementary Figure 4. The spatial association statistic between the adult walnut trees and the current year seedlings, cross-type L(r), evaluated at a range of distances r for each plot of the squirrel-mouse and squirrel-only site. When cross-type L(r) (continuous line) is outside the confidence interval (with a risk of $\alpha = 0.05$; greyscale), it indicates there is evidence against the null model (heterogeneous Poisson pattern; dotted line). Measured values below the confidence interval indicate a repulsive relationship, and values above the interval indicate an attractive relationship.

Although all plots at the squirrel-only site showed an attractive spatial relationship between seedlings and adult trees more than 20 m in distance r, the departure from independence between seedlings and adult trees was not always significant in all plots of the squirrel-only and squirrel-mouse sites.

Supplementary Results 2

Supplementary Table 1. Summary of camera trapping-based hit rates (videos/camera-day) for red squirrels and live trapping-based density estimates (individuals/ha) for wood mice.

Site	Plot	Hit rates of red squirrels (videos/camera-day)	Density of wood mice (individuals/ha)
Squirrel-mouse	1	0.0370	27.0
	2	0.0247	32.0
Squirrel-only	1	0.0370	-
	2	0.0556	-

A total of 8 videos (range 0 to 3 videos per day) of red squirrels were obtained in 202.5 camera days of filming effort. The filtering effort for the camera trapping session ranged from 40.5 to 54 camera days, where 1 camera day represents one functioning camera deployed for 24 h. The estimated hit rates of red squirrels in each plot during the camera trapping session ranged from 0.025 to 0.056 videos/camera days.

A total of 59 wood mice (range 2 to 18 individuals per day) were captured in live trapping sessions. The estimated density of wood mice in each plot during the live trapping session ranged from 27.0 to 32.0 individuals/ha.

Supplementary Results 3

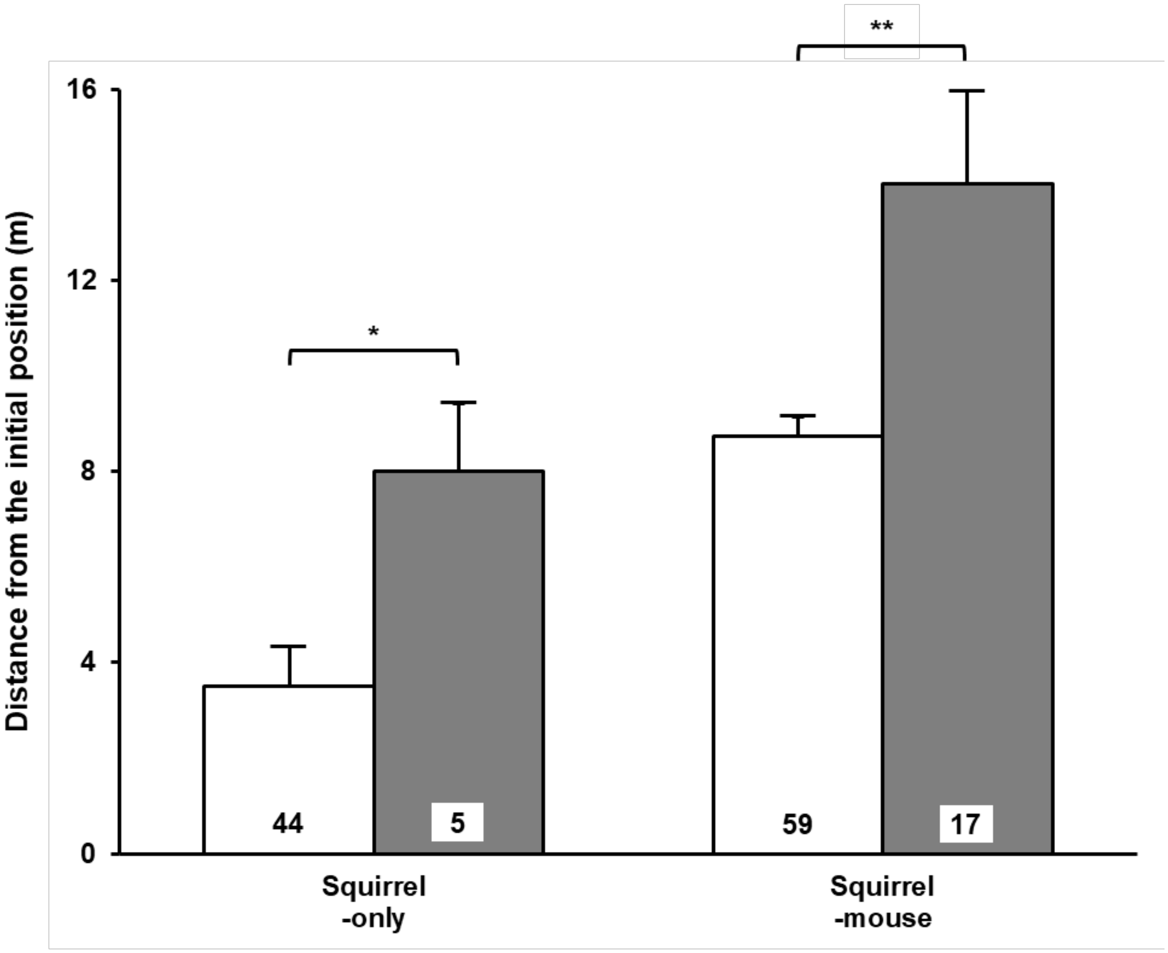

***$p < 0.001$, **$p < 0.01$, *$p < 0.05$

Supplementary Figure 5. Distance from the initial position of magnet-attached walnut seeds (filled bars) and only magnets (blank bars) to the relocated position at the squirrel-only and squirrel-mouse site. The figure shows the mean ± 1 SE. * indicates significant difference, based on Mann–Whitney U test. Sample sizes are shown inside the bars.

Supplementary References

1. Wiegand, T. & A. Moloney, K. Rings, circles, and null - models for point pattern analysis in ecology. *Oikos* **104**, 209-229 (2004).
2. Perry, G. L., Miller, B. P. & Enright, N. J. A comparison of methods for the statistical analysis of spatial point patterns in plant ecology. *Plant Ecol.* **187**, 59-82 (2006).
3. Ripley, B. Modeling Spatial Patterns. *Journal of Royal Statistical Society, Series B (Methodological)* **39**, 172–212 (1977).
4. Baddeley, A. & Turner, R. spatstat: An R Package for Analyzing Spatial Point Patterns. *J. Stat. Softw.* **12**, 1548–7660 (2005).
5. Hanisch, K. H. & Stoyan, D. Formulas for the second-order analysis of marked point processes. *Statistics: A Journal of Theoretical and Applied Statistics* **10**, 555–560 (1979).
6. Wiegand, T., Gunatilleke, S. & Gunatilleke, S. Species associations in a heterogeneous Sri Lankan dipterocarp forest. *Am. Nat.* **170**, 77–95 (2007).
7. Goreaud, F., Courbaud, B. & Collinet, F. Spatial structure analysis applied to modelling of forest dynamics: a few examples. In Proceedings of the IUFRO workshop, Empirical and process based models for forest tree and stand growth simulation (pp. 20-26) (1997).
8. Goreaud, F. & Pélissier, R. Avoiding misinterpretation of biotic interactions with the intertype K12 - function: population independence vs. random labelling hypotheses. *J. Veg. Sci.* **14**, 681-692 (2003).
9. Villette, P., Krebs, C. J. & Jung, T. S. Evaluating camera traps as an alternative to live trapping for estimating the density of snowshoe hares (*Lepus americanus*) and red squirrels (*Tamiasciurus hudsonicus*). *Eur. J. Wildl. Res.* **63**, 7 (2017).
